# Pressure-induced Jahn–Teller switch in the homoleptic hybrid perovskite [(CH_3_)_2_NH_2_]Cu(HCOO)_3_: orbital reordering by unconventional degrees of freedom†

**DOI:** 10.1039/d1tc01966j

**Published:** 2021-06-09

**Authors:** Rebecca Scatena, Michał Andrzejewski, Roger D. Johnson, Piero Macchi

**Affiliations:** Department of Physics, Clarendon Laboratory, University of Oxford, Parks Road Oxford OX1 3PU UK rebecca.scatena@physics.ox.ac.uk; Department of Chemistry and Biochemistry, University of Bern, Freiestrasse 3 Bern CH-3012 Switzerland; Department of Physics and Astronomy, University College London, Gower Street London WC1 6BT UK; Department of Chemistry, Materials, and Chemical Engineering, Polytechnic of Milan, Via Mancinelli 7 Milan 20131 Italy

## Abstract

Through *in situ*, high-pressure X-ray diffraction experiments we have shown that the homoleptic perovskite-like coordination polymer [(CH_3_)_2_NH_2_]Cu(HCOO)_3_ undergoes a pressure-induced orbital reordering phase transition above 5.20 GPa. This transition is distinct from previously reported Jahn–Teller switching in coordination polymers, which required at least two different ligands that crystallize in a reverse spectrochemical series. We show that the orbital reordering phase transition in [(CH_3_)_2_NH_2_]Cu(HCOO)_3_ is instead primarily driven by unconventional octahedral tilts and shifts in the framework, and/or a reconfiguration of A-site cation ordering. These structural instabilities are unique to the coordination polymer perovskites, and may form the basis for undiscovered orbital reorientation phenomena in this broad family of materials.

## Abstract 2

Unconventional octahedral tilts unique to the coordination polymer perovskites support a high-pressure structural phase transition in [(CH_3_)_2_NH_2_]Cu(HCOO)_3_, which results in Jahn–Teller axis directional switching and orbital reordering.

## Graphical abstract

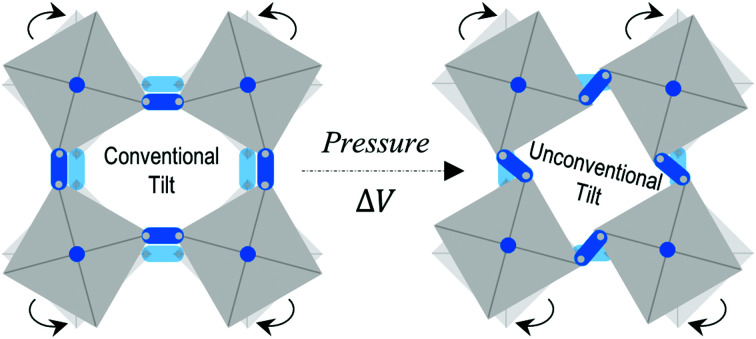

## Introduction

Transition metal perovskites of general chemical formula ABX_3_ are a compositionally rich family of materials in which a strong interplay between charge, orbital and magnetic degrees of freedom is manifest. In some cases, this interplay leads to technologically significant materials properties such as superconductivity and colossal magneto-resistance.^1–3^ Orbital ordering in particular affords an efficient mechanism for coupling the transition metal's electronic degrees of freedom to the crystal lattice *via* the Jahn–Teller (JT) effect,^4^ and hence is key to our understanding, development, and exploitation of structure–property relationships in these materials. For example, in the case of magnetic order the nature and strength of magnetic superexchange interactions between metal ions is regulated by the ordered spatial orientation of d-orbitals,^5–7^ and in the case of charge ordering, the local electronic configuration of cations is directly coupled to orbital occupation and the respective orbital order. Hence, the ability to tune and control orbital order may provide a handle on multiple interacting electronic degrees of freedom. Given the strong coupling between orbital order and the crystal structure it is natural to attempt control of orbital order by applied hydrostatic pressure or strain–with hydrostatic pressure being advantageous with regards to mechanical stability and its applicability to polycrystalline ceramics that are commonplace in technology. In this context, the role of strain and pressure in tuning JT physics has been discussed in theory.^8,9^

A canonical example of structure–property relationships established through orbital ordering is found in the fully inorganic transition metal perovskite KCuF_3_. In this compound the orbital ordering scheme can be appreciated by the alternating position of JT axes of the Cu^2+^ ions, which directly determines the A-type magnetic structure of ferromagnetic (FM) **ab**-plane layers coupled antiferromagnetically (AFM) along the **c**-axis,^1^ as predicted by the Goodenough–Kanamori–Anderson (GKA) rules.^5–7^ Neutron diffraction experiments performed on KCuF_3_ under high pressure showed that in the limit of quasi-hydrostatic compression from ∼0 to 8 GPa, all Cu–F bond lengths progressively shorten.^10^ However, the JT elongated Cu–F bonds are compressed by almost 0.18(1) Å, whereas the equatorial Cu–F bonds only by 0.04(1) Å. This anisotropic response of the Cu–F bonds to hydrostatic compression leads to a considerable suppression of the JT distortion, possibly restoring the degeneracy between e_g_ orbitals at higher pressure. Whilst this experiment clearly demonstrated tuning of the orbital state in KCuF_3_, it did not demonstrate any dramatic reconfiguration of the orbital order.

**Fig. 1:**
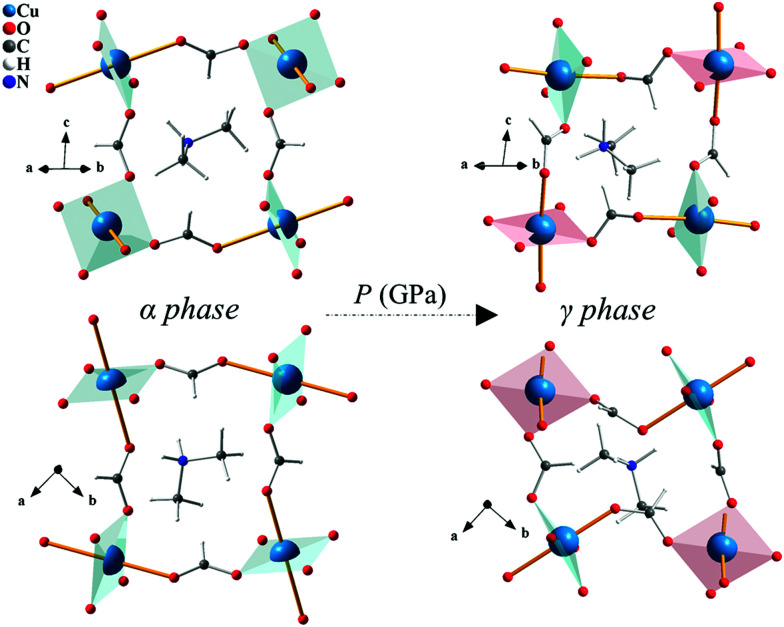
Crystal structure of the α-phase at ambient pressure and γ-phase at 9.1 GPa. The equatorial planes are drawn in blue and red. JT axis are shown as yellow sticks.

In this article, we have identified a homoleptic coordination polymer structural analogue of KCuF_3_; dimethylammonium copper formate, [(CH_3_)_2_NH_2_]Cu(HCOO)_3_ (albeit with additional conventional octahedral tilts and rotations). Here, the A site K^+^ ions are replaced by dimethylammonium counter cations [(CH_3_)_2_NH_2_]^+^, and the X site F^−^ ions are replaced by formate anions (HCOO)^−^. Both substitutions are homovalent, hence preserving the Jahn–Teller active 2+ oxidation state of copper at the B sites. At ambient pressure [(CH_3_)_2_NH_2_]Cu(HCOO)_3_ adopts a monoclinic *I*2/*a* structure (here denoted α-phase), with B site Cu^II^ cations bridged by formate anions in *anti*–*anti* fashion along all three directions, forming centrosymmetric axially elongated CuO_6_ octahedra arranged in a conventional (a^−^a^−^c^−^) tilt pattern (in Glazer notation).^11^ The axial (JT elongated) bonds alternate between subsequent Cu^II^ ions along the **a** + **b** and **a** − **b** crystallographic directions, whereas only equatorial bonds are pointing along **c** (Fig. 1). Therefore, at ambient pressure [(CH_3_)_2_NH_2_]Cu(HCOO)_3_ has the same orbital order as found in KCuF_3_, and consequently the two systems support the same A-type magnetic structure at low temperature.^12^ However, coordination polymers that adopt a perovskite-like framework have additional structural degrees of freedom associated with A-site ordering and unconventional octahedral tilts and columnar shifts.^13^ Based on these key differences, one might anticipate significantly different behaviour under applied hydrostatic pressure.

## Experimental

### High-pressure crystallography

Single crystals of [(CH_3_)_2_NH_2_]Cu(HCOO)_3_ were loaded in a Merrill–Bassett diamond-anvil cell (DAC)^14^ (Fig. S1 ESI†). A mixture of MeOH : EtOH (4 : 1 volume ratio) was used as pressure-transmitting medium.^15^ The pressure inside the DAC was calibrated using a ruby fluorescence method.^16,17^ The experiment was reaped on two crystals at room temperature. Single-crystal diffraction experiments for loading A were carried out in house on an Oxford Diffraction SuperNova area-detector diffractometer employing mirror optics, monochromated, and Al filtered^18^ microsource Mo K_α_ radiation (*λ* = 0.71073 Å). Single-crystal diffraction experiments for loading B were performed at the X04SA Material Science beamline of Swiss Light Source (Paul Scherrer Institute, Switzerland) with a Pilatus 6M detector (*λ* = 0.49647 Å).^19^ The crystal was gradually compressed up to about 9 GPa. Data collection and reduction were performed by using CrysAlisPro software.^20^ As the hydrostatic limit of the mixture was reached, the pressure inside the DAC was decreased to 7 GPa. Two pressure points, before and after the transition, were remeasured using synchrotron radiation to determine more accurate atomic positions, at 4.75 and 8.3 GPa, respectively. Crystal structures were solved by using SHELXT^21^ and the spherical independent atom model (IAM) was refined with SHELXL,^22^ as implemented in Olex2 interface.^23^ Selected crystallographic data and refinement details are reported in Tables S1 and S2 (ESI†).

### Mode decomposition analysis

The tetragonal *P*4/*mmm* [*a* = 6.2514 Å, *c* = 5.7444 Å] hypothetical high-symmetry parent cell was used to derive the irreducible representation constituting the low-symmetry structures (Table S3 ESI†). A structural CIF file of the parent cell with space-group symmetry, cell parameters and the positions of the atoms excluding the hydrogen atoms was imported in ISODISTORT^24^ to derive, with “method 4”, the mode decomposition of the distorted structures *i.e.* the monoclinic *I*2/*a* α-phase and triclinic *P*1̄ γ-phase. The basis (1,−1,0)(1,1,0)(0,0,2) was applied to transform between parent and distorted cells, and obtain the lattice parameters of the undistorted supercell: *a* = 8.84082, *b* = 8.84082, *c* = 11.48882, alpha = 90.0, beta = 90.0, gamma = 90.0 used in the definition of the modes. The PCR file produced by ISODISTORT includes the description of the modes through the polarization vectors of the displacive symmetry modes for each atom and the refinable amplitudes of the displacive symmetry modes. The refinement of amplitudes and isotropic thermal parameters against the experimental structure factor was carried out using Fullprof.^25^ The refined mode amplitudes were converted into absolute displacements in Å through normalization factor and the polarization vector direction (Tables S4 and S5 ESI†). The irreducible representations derived for the tetragonal to monoclinic and tetragonal to triclinic distortions are shown, respectively, in Fig. S5 and S6 (ESI†).

## Results and discussion

We performed high-pressure single-crystal X-ray diffraction measurements using diamond anvil cells loaded with a single crystal sample of [(CH_3_)_2_NH_2_]Cu(HCOO)_3_ and a mixed MeOH : EtOH pressure transmitting medium. The experiments were carried out in the range 0–9 GPa using both laboratory-based Mo K_α_ radiation and synchrotron radiation (*λ* = 0.49647 Å). The α-phase was found to be stable up to 5.20 GPa (Table 1). Throughout this low pressure phase the Cu–O coordinative bonds were found to be the most compressible bonds in the structure, and by 5.20 GPa the axial and equatorial bonds were shortened by 0.207(13) Å and 0.036(8) Å, respectively–consistent with the pressure induced anisotropic octahedral compression found in KCuF_3_.^10^

**Table 1:** Pressure variation of the unit-cell in [(CH_3_)_2_NH_2_]Cu(HCOO)_3_ from single crystal X-ray diffraction at 295 K

P/GPa	0.0001	1.0	2.23	3.40	4.75	5.20	7.15	8.3	9.1
SG	*I*2/*a*	*I*2/*a*	*I*2/*a*	*I*2/*a*	*I*2/*a*	*I*2/*a*	*P*1̄	*P*1̄	*P*1̄
*a*/Å	8.8330(4)	8.6895(13)	8.5019(14)	8.323(3)	8.2878(9)	8.086(4)	7.2638(16)	7.2432(13)	7.2332(8)
*b*/Å	8.7093(4)	8.5925(10)	8.5043(10)	8.4733(14)	8.4645(6)	8.466(2)	8.5726(15)	8.5444(11)	8.5452(10)
*c*/Å	11.4145(5)	11.3323(4)	11.2256(4)	11.1183(7)	11.0942(3)	10.9541(9)	11.2929(17)	11.2169(19)	11.085(4)
*α*/°	90	90	90	90	90	90	92.384(13)	92.441(13)	92.908(17)
*β*/°	96.224(4)	95.623(7)	95.055(7)	94.553(12)	94.521(4)	94.215(17)	101.797(16)	102.055(15)	102.278(18)
*γ*/°	90	90	90	90	90	90	91.352(16)	91.298(13)	91.268(10)
*V*/Å^3^	872.93(7)	842.05(16)	808.48(17)	781.7(3)	775.86(10)	747.8(4)	687.4(2)	677.92(19)	668.2(3)

**Fig. 2:**
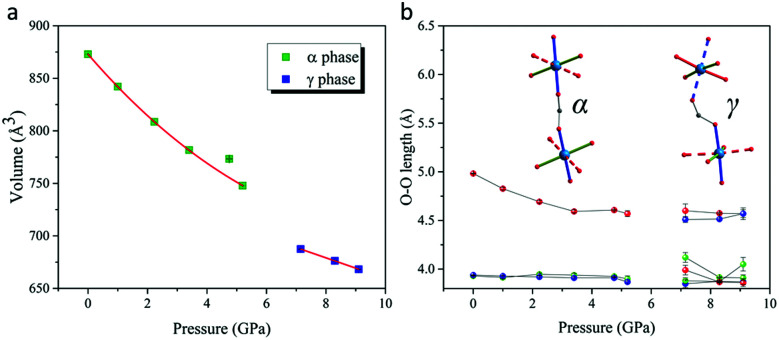
(a) Volume and (b) dimensions of coordination octahedra (O–O length) as a function of pressure. JT axes are shown as dotted sticks.

Analysis of diffraction data collected at 7.15 GPa showed a dramatic departure of the α and γ angles from 90° and a breaking of the *I*-centering translational symmetry through emergence of reflection intensities with *h* + *k* + *l* = 2*n* + 1 where *n* is an integer (Fig. S2 ESI†). Together, these observations revealed a structural phase transition in the region 5.20 → 7.15 GPa from monoclinic *I*2/*a* to a triclinic *P*1̄ supercell (Table 1), accompanied by a significant volume contraction as shown in Fig. 2a. We label this high pressure phase the γ-phase, which we note is metrically different from the *P*1̄ phase of [(CH_3_)_2_NH_2_]Cu(HCOO)_3_ previously discovered at 5.96 GPa by Collings and co-workers (the β-phase).^26^

Structural refinements against the 7.15 GPa data showed that the axial (JT elongated) Cu–O bonds initially along the **a** + **b** direction in the α-phase switched to lie along the **c**-axis in the γ-phase, while axial bonds initially along the **a** − **b** direction maintained their orientation (Fig. 1). This dramatic structural switch preserved *anti*–*anti* bonding of formate along the **a** + **b** direction (now involving only equatorial Cu–O bonds), but is accompanied by *syn*–*anti* bonding of formate in all other directions (Fig. 1 and 2b). Importantly, this directional switch of the JT axes can be understood in terms of a reordering of the Cu^II^ d-orbitals depicted by the equatorial planes drawn in Fig. 1 (see also Fig. S3 and S4 in ESI†). The GKA rules have been shown to correctly predict the ambient-pressure A-type magnetic ground state of [(CH_3_)_2_NH_2_]Cu(HCOO)_3_,^12^ in which Cu^II^ ions form FM layers extending in the **ab** plane, which stack antiferromagnetically along **c**. If we apply the same GKA rules for formate-mediated exchange (as outlined in ref. 12) to the high-pressure γ-phase described above, one predicts strong AFM superexchange pathways along **a** + **b**, and weak FM superexchange pathways along **c** and **a** − **b**; *i.e.* a different A-type magnetic structure in which the AFM stacking direction has switched from being along **c** in the α-phase to **a** + **b** in the γ-phase.

Pressure induced orbital reordering due to a JT switch has been observed in Cr^II^ and Cu^II^ Tutton salts^27,28^ and in a Mn_12_ single-molecule magnet cluster.^29^ Such transitions have also been found in a relatively small number of coordination polymers composed of at least two different ligand species that disobey the spectrochemical series in the low-pressure phase, hence enabling the different ligand strengths to act as the driving force behind the phase transition.^30–33^ Interestingly, in these coordination polymers a dramatic change in the magnetic structure was caused by the orbital reordering,^30,32,34–36^ in analogy with our predictions for [(CH_3_)_2_NH_2_]Cu(HCOO)_3_. To the best of our knowledge, pressure induced orbital reordering due to a JT switch has never been observed in a homoleptic coordination polymer. Hence, this observation points towards an altogether novel mechanism for orbital reordering that may apply to a much wider family of materials.

**Fig. 3:**
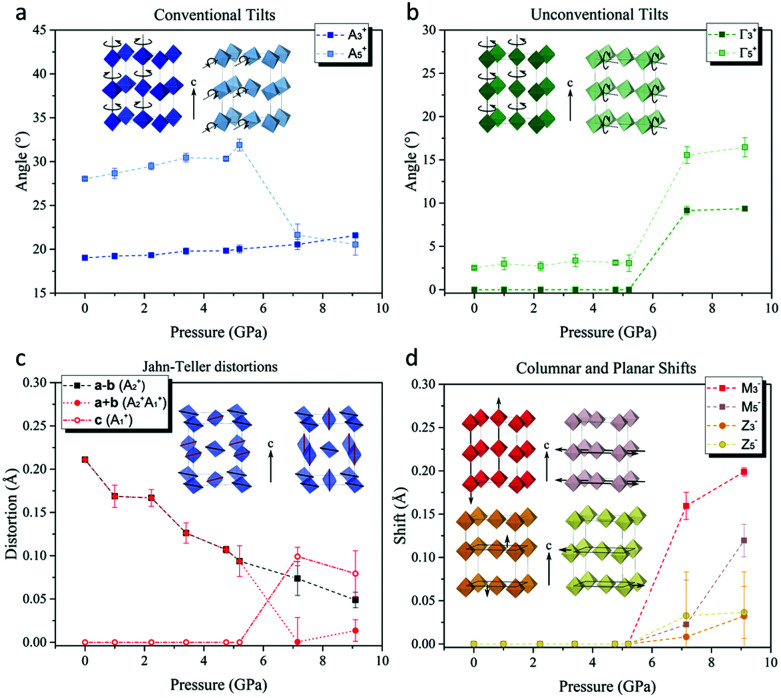
Pressure dependence of (a) conventional octahedra tilts rotation angles, (b) unconventional octahedra tilts rotation angles, (c) Jahn–Teller distortions and (d) columnar and planar shifts of the framework.

To untangle the underlying structural mechanism responsible for orbital reordering in [(CH_3_)_2_NH_2_]Cu(HCOO)_3_ we refined the crystal structure in terms of symmetry adapted displacive modes defined with respect to a hypothetical high-symmetry parent common to both α- and γ-phases. The adopted *P*4/*mmm* parent allows decomposing structural distortions relevant to this class of materials (*e.g.* JT distortion and octahedra tilts) into independent symmetry adapted modes. Moreover, compared to the standard choice *Pm*3̄*m*, *P*4/*mmm* symmetry has the advantage of describing all possible structural distortion, including those pertinent to [(CH_3_)_2_NH_2_]^+^ cations, exclusively in terms of displacive modes without having to include occupational modes.^24^ The active distortion modes of all relevant Wyckoff sites were identified by decomposing the experimental α- and γ-phases using ISODISTORT,^24^ and classified according to the irreducible representation by which they transformed. The α-phase decomposes into modes of three *Γ*-point irreducible representations (*Γ*_1_^+^, *Γ*_4_^+^, and *Γ*_5_^+^) and modes of three *A*-point irreducible representations (*A*_2_^+^, *A*_3_^+^, and *A*_5_^+^). The largest distortions of the {Cu(HCOO)_3_^−^}_*n*_ perovskite framework were associated with the (*a*^−^*a*^−^*c*^−^) octahedral tilts, which transform by *A*_3_^+^ and *A*_5_^+^ (Fig. 3a). These modes alone establish the *I*2/*a* α-phase symmetry and as such they can be considered primary modes with respect to our hypothetical parent. The Jahn–Teller distortions transform by *A*_2_^+^, and can be considered secondary modes–that is to say they are compatible with the crystal symmetry established by the octahedral tilts.

Interestingly, the *I*2/*a* crystal symmetry also allows unconventional octahedral tilts (uniform rotations about the *I*2/*a***b**-axis, Fig. 3b) that transform by *Γ*_5_^+^, however the respective mode amplitudes are negligible in the α-phase. These unconventional tilts would result in a departure from *anti*–*anti* type bonding of every formate anion, towards a *syn*–*anti* configuration. The fact that these distortions are allowed by symmetry yet found to be small implies the presence of microscopic interactions that energetically favour *anti*–*anti* bonding over *syn*–*anti* bonding in the perovskite framework, as reported in the study by H. L. Bostrom,^37^ thus prompting the structure to adopt conventional octahedra tilts only.

The amplitude of conventional octahedral rotations about the **c**-axis (*A*_3_^+^) varied little over the measured pressure range (Fig. 3a), while the conventional octahedral rotations about an axis lying perpendicular to the **c**-axis (*A*_5_^+^) are reduced in amplitude upon entering the γ-phase. Furthermore, their axis of rotation, constrained by symmetry to be parallel to the **a**-axis in the α-phase, now rotates by about 35° towards the **b**-axis. Throughout the α-phase one observes a significant, monotonic suppression of the JT distortion, as mentioned previously (Fig. 3c). In the γ-phase, new JT displacive modes that transform by the *A*_1_^+^ irreducible representation admix with the α-phase JT displacements (*A*_2_^+^), leading to the observed JT switch.

The number of modes that enter into the decomposition of the γ-phase structure is considerably greater than those appearing in the decomposition of the α-phase (see ESI†). In further contrast to the α-phase, our analysis showed that the low symmetry of the γ-phase can only be reached by no less than three primary, symmetry breaking modes. The possible combinations of primary modes is vast, yet all include either *M*-point or *Z*-point modes that do not appear in the decomposition of the α-phase. The relevant *M*-point primary modes transform by either the *M*_2_^−^, *M*_3_^−^, or *M*_5_^−^ irreducible representations, and correspond to the ordering of A site dimethylammonium molecular orientations, unconventional **c**-axis columnar shifts parallel to **c**, and unconventional **c**-axis columnar shifts perpendicular to **c**, respectively (Fig. 3d). The relevant *Z*-point primary modes transform by either *Z*_3_^−^ or *Z*_5_^−^, and correspond to **ab**-plane planar shifts parallel and perpendicular to **c**, respectively. We also note that the *Γ*_5_^+^ unconventional octahedral rotations, allowed by symmetry in the α-phase but found to have negligible amplitudes, now appear with large amplitude in the γ-phase (Fig. 3b).

It is well established that the unit cell volume of perovskite-type structures can be reduced through a linear-quadratic coupling of the lattice strain to conventional octahedra tilts. [(CH_3_)_2_NH_2_]Cu(HCOO)_3_ already hosts large conventional tilts at ambient pressure, and, somewhat surprisingly, the amplitude of these tilts varies little with increasing pressure (Fig. 3a). Instead, the volume contraction in the α-phase largely originates in a compression of the coordinative bonds (Fig. 3c, discussed above). Unconventional octahedra tilts and shifts can couple to lattice strain in the same way as the conventional octahedral tilts, hence they represent extra structural degrees of freedom by which the unit cell volume can be reduced. Since pressure stabilises structural deformations that lead to unit cell volume reduction, we suggest that unconventional octahedra tilts and shifts naturally play a leading role in the pressure-induced phase transition and associated Jahn–Teller switch. This scenario is further supported by the absence, within the same pressure regime, of a phase transition in KCuF_3_, in which the network of corner sharing octahedra forbid such unconventional structural degrees of freedom. All unconventional distortions introduce energetically unfavourable *syn*–*anti b*onding of the formate anions, hence the critical behaviour associated with the phase transition might be understood in terms of an energetic competition between formate bonding requirements and the steric contraction of the lattice. Finally, we note that the [(CH_3_)_2_NH_2_]^+^ cations at the A sites interact weakly with each other, and also weakly with the rest of the perovskite framework, hence the reconfiguration of their order is unlikely to drive the large structural distortions observed at the phase transition.

## Conclusions

In summary, we have showed that *quasi*-hydrostatic compression in the region 5.20 → 7.15 GPa induced a structural phase transition in single-crystals of [(CH_3_)_2_NH_2_]Cu(HCOO)_3_–a model perovskite-like coordination polymer. At the transition the crystal symmetry was lowered from monoclinic to a previously unobserved triclinic phase in which half of the Cu^II^ Jahn–Teller axes had switched from the **a** + **b** to the **c** crystallographic direction. Such orbital reordering is unexpected in homoleptic coordination polymers and, to the best of our knowledge, has never been observed before. Symmetry analysis demonstrated that the phase transition is primarily driven by unconventional octahedral tilts and shifts in the framework, and/or a reconfiguration of A-site cation ordering. These structural instabilities are unique to the coordination polymer perovskites, which naturally explains the absence of a high-pressure phase transition in the canonical inorganic analogue KCuF_3_, and implies that such transitions may occur in a wide family of coordination polymers of current scientific interest. The unconventional distortions require a departure from energetically favourable *anti*–*anti* bonding of formate ligands towards the *syn*–*anti* limit. We suggest that this energetic cost competes with the steric contraction of the lattice, thereby resulting in a structural phase transition under pressure. Our results serve as a proof of principle, demonstrating that it is possible to control orbital order in coordination polymer perovskites by applied hydrostatic pressure. Importantly, the pressure induced switch of the Jahn–Teller axis is expected to reconfigure the main magnetic interactions in the material, hence magnetism and magnetic order under high pressure presents an interesting topic for future studies of [(CH_3_)_2_NH_2_]Cu(HCOO)_3_ and related materials.

## Author contributions

R. S. and P. M. contributed to conceptualisation and resources. Supervision was provided by P. M. and R. D. J., while R. S. administered the project. P. M. and R. S. acquired funding for the project. M. A. and R. S. carried out the investigation. M. A., R. S. and R. D. J. carried out the formal analysis of the data. R. S. and R. D. J. wrote the manuscript.

## Conflicts of interest

There are no conflicts to declare.

## Supplementary Material

TC-009-D1TC01966J-s001

TC-009-D1TC01966J-s002
