# Supplementary material for: Pressure-induced Jahn–Teller switch in the homoleptic hybrid perovskite [(CH3)2NH2]Cu(HCOO)3: orbital reordering by unconventional degrees of freedom
Source: J Mater Chem C Mater. 2021 Jun 9;9(25):8051–6. doi: 10.1039/d1tc01966j (PMC8246535; doi:10.1039/d1tc01966j)
Supplement: TC-009-D1TC01966J-s001 [file TC-009-D1TC01966J-s001.pdf]

Electronic Supplementary Material (ESI) for Journal of Materials Chemistry C.
This journal is © The Royal Society of Chemistry 2021

Pressure-induced Jahn-Teller Switch in the Homoleptic Hybrid Perovskite $[(CH_3)_2NH_2]Cu(HCOO)_3$: Orbital Reodering by Unconventional Degrees of Freedom

Rebecca Scatena,*[a] Michał Andrzejewski,[b] Roger D. Johnson[c] and Piero Macchi[d]

[a] Department of Physics, Clarendon Laboratory, University of Oxford, Parks Rd., Oxford OX1 3PU, United Kingdom

[b] Department of Chemistry and Biochemistry, University of Bern, Freiestrasse 3, Bern CH-3012, Switzerland

[c] Department of Physics and astronomy, University College London, Gower Street, London WC1 6BT, United Kingdom

[d] Department of Chemistry, Materials, and Chemical Engineering, Polytechnic of Milan, Via Mancinelli 7, Milan 20131, Italy

Supplementary Information

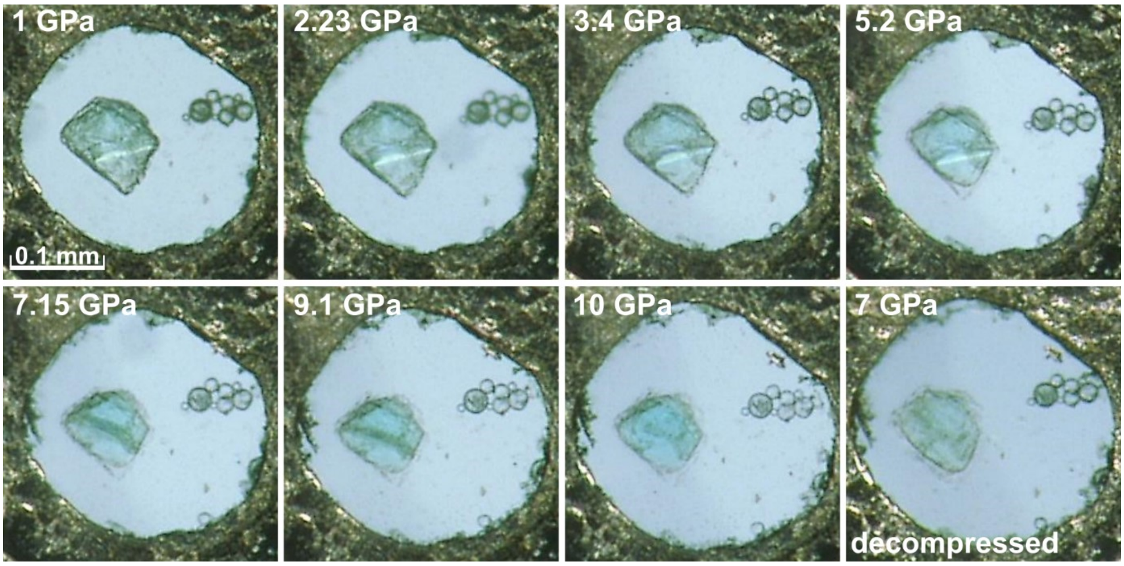

Figure S1. Single crystal of [Cu(HCOO)3]H2N(CH3)2 in DAC. Ruby spheres are the spheres located by the edge.

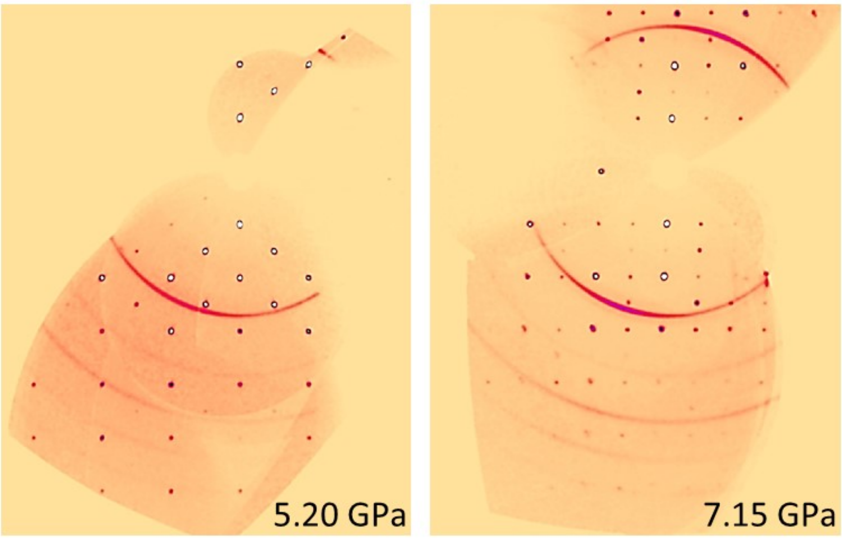

Figure S2. (0kl) diffraction plane for a single crystal of $[(CH_3)_2NH_2]Cu(HCOO)_3$ at 5.20 and 7.15 GPa.

Table S1. Selected single crystal diffraction data and refinements details for $[(CH_3)_2NH_2]Cu(HCOO)_3$ at 295 K.

Pressure (GPa)	0.00001 GPa	1.0	2.23	3.40	3.8
Loading	-	A	A	A	B
Crystal size	0.6x0.3x0.3	0.1x0.08x0.05	0.1x0.08x0.05	0.1x0.08x0.05	0.11x0.0.072x0.0 0.056
phase	α	α	α	α	α
Crystal system	monoclinic	monoclinic	monoclinic	monoclinic	monoclinic
Space group	*I*2/*a*	*I*2/*a*	*I*2/*a*	*I*2/*a*	*I*2/*a*
λ (Å)	0.71073	0.71073	0.71073	0.71073	0.49647
a (Å)	8.8330(4)	8.6895(13)	8.5019(14)	8.323(3)	8.2878(9)
b (Å)	8.7093(4)	8.5925(10)	8.5043(10)	8.4733(14)	8.4645(6)
c (Å)	11.4145(5)	11.3323(4)	11.2256(4)	11.1183(7)	11.0942(3)
α (°)	90	90	90	90	90
β (°)	96.224(4)	95.623(7)	95.055(7)	94.553(12)	94.521(4)
γ (°)	90	90	90	90	90
Volume (Å^3)	872.93(7)	842.05(16)	808.48(17)	781.7(3)	775.86(10)
Z / Z'	4/1	4/1	4/1	4/1	4/1
Min/max hkl	-11/12, -11/12, -15/15	-6/5, -8/5, -14/8	-5/5, -8/5, -8/13	-4/5, -8/6, -8/13	-7/9, -9/10, -17/17
2θ ranges (°)	7.182 – 59.158	6.684 – 52.856	6.79 – 51.294	6.052 – 51.864	5.152 – 52.944
Collected/unique	8550 / 1133	838 / 254	771 / 237	710 / 229	1139 / 688
r_{int} / %	4.32	3.21	2.87	4.05	1.57
Completeness (%)	99.6	29.26	30.50	29.78	29.28
GOF *w*	1.140	1.115	1.103	1.108	1.091
R_1(F) % [I ≥2σ]	2.36	3.39	3.35	4.38	3.59
R_1(F) % [all]	2.89	4.29	4.36	5.24	4.15
w_1/w_2	0.03 / 0.40	0.06 / 1.40	0.033 / 5.91	0.06 / 1.54	0.082 / 0
peak/hole (eÅ^{-3})	0.32 / -0.58	0.25 / -0.28	0.23 / -0.26	0.34 / -0.33	0.39 / -0.41

Table S2. Selected single crystal diffraction data and refinements details for $[(CH_3)_2NH_2]Cu(HCOO)_3$ at 295 K.

Pressure (GPa)	5.20	7.15	8.30	9.10
Loading	A	A	B	A
Crystal size	0.1x0.08x0.05	0.1x0.08x0.05	0.11x0.072x0.056	0.1x0.08x0.05
phase	α	γ	γ	γ
Crystal system	monoclinic	triclinic	triclinic	triclinic
Space group	*I*2/*a*	$P\bar{1}$	$P\bar{1}$	$P\bar{1}$
λ (Å)	0.71073	0.71073	0.49647	0.71073
a (Å)	8.086(4)	7.2638(16)	7.2432(13)	7.2332(8)
b (Å)	8.466(2)	8.5726(15)	8.5444(11)	8.5452(10)
c (Å)	10.9541(9)	11.2929(17)	11.2169(19)	11.085(4)
α (°)	90	92.384(13)	92.441(13)	92.908(17)
β (°)	94.215(17)	101.797(16)	102.055(15)	102.278(18)
γ (°)	90	91.352(16)	91.298(13)	91.268(10)
Volume (Å^3)	747.8(4)	687.4(2)	677.92(19)	668.2(3)
Z / Z'	4/1	4/2	4/2	4/2
Min/max hkl	-4/4, -7/5, -13/7	-6/6, -10/7, -15/15	-12/12, -14/15, -12/9	-8/4, -7/6, -4/7
2θ ranges (°)	6.088 – 50.962	3.688 – 56.738	3.334 – 55.312	4.776 – 51.684
Collected/unique	350 / 120	3829 / 737	3954 / 2430	1301 / 432
r_{int} / %	2.45	5.96	6.41	4.38
Completeness (%)	17.19	21.38	47.1	17.69
GOF *w*	1.115	1.063	1.078	1.118
R_1 (F) % [I ≥2σ]	3.36	7.67	5.08	5.47
R_1 (F) % [all]	3.95	13.06	10.64	8.66
w_1/w_2	0.061 / 10.3	0.142 / 10.3	0.055 / 0	0.095 / 1.32
peak/hole (eÅ^{-3})	0.19 / -0.22	0.67 / -0.66	0.7 / -0.5	0.34 / -0.31

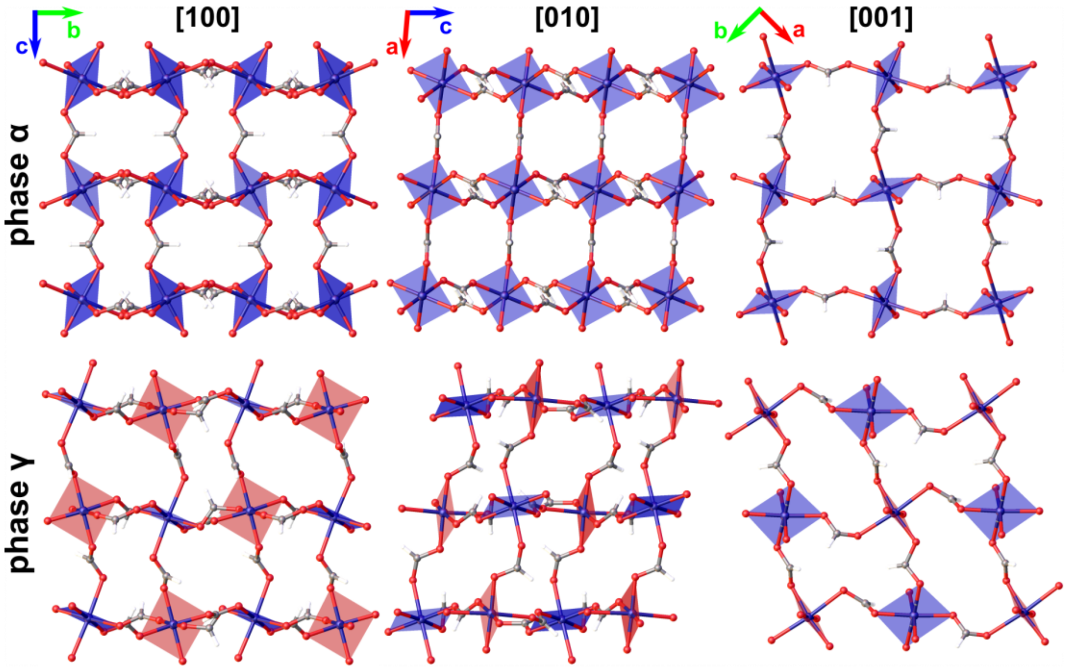

Figure S3. Fragments of $[(CH_3)_2NH_2]Cu(HCOO)_3$ structure in the α and γ phase viewed along the main crystallographic directions. Equatorial planes perpendicular to JT axes are highlighted by the blue and red square planes.

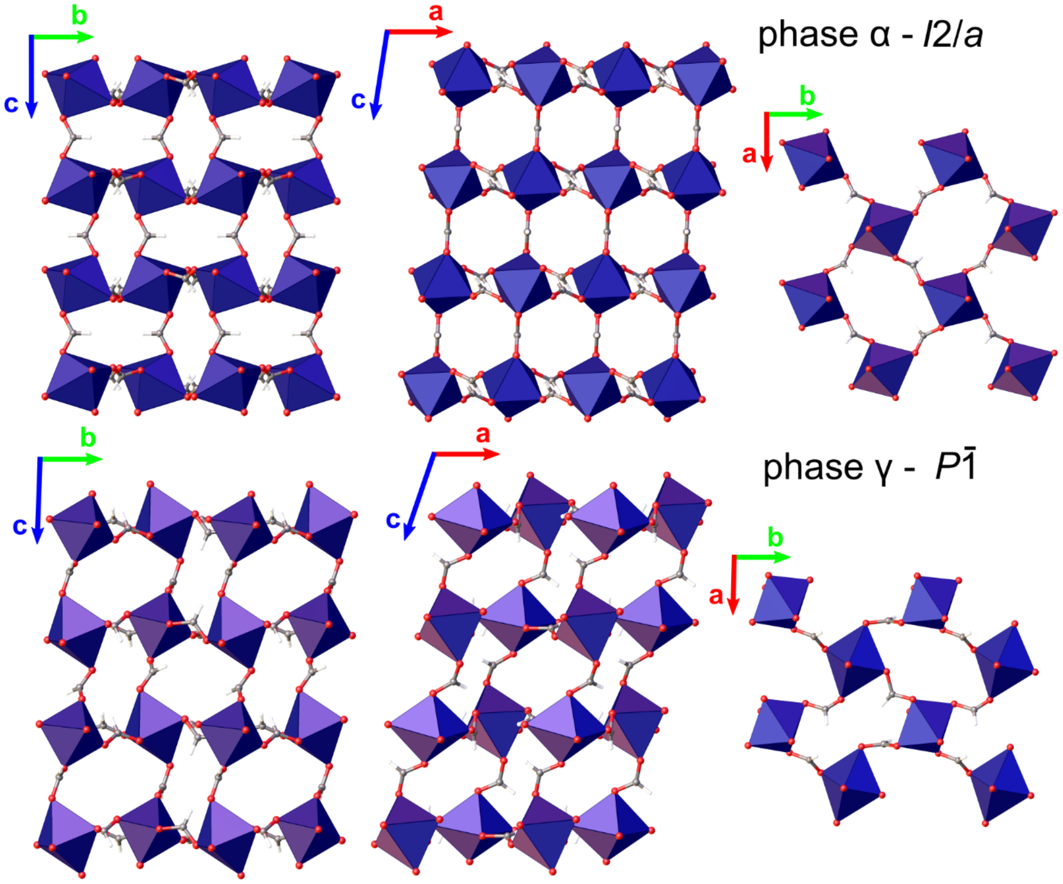

Figure S4. View along the *a*, *b*, and *c* crystallographic axis of the CuO_6 octahedra bridged by formate $[HCOO^-]$ ligands in the α and γ phases. The dimethylammonium $[H_2N(CH_3)_2]^+$ A site cations were omitted for sake of clarity.

Table S3. Structure of the *P*4/mmm parent cell.

Atom	x	y	z	g
Cu1	0.000000	0.000000	0.000000	0.0625
O1	0.000000	0.000000	0.305817	0.1250
O2	0.321708	0.000000	0.000000	0.2500
C1	0.500000	0.000000	0.000000	0.1250
C2	0.000000	0.000000	0.500000	0.0625
C3	0.500000	0.500000	0.597350	0.1250
N1	0.500000	0.500000	0.500000	0.0625

Table S4. Mode amplitudes refined as function of pressure for the monoclinic *I*2/a α-phase from single crystal x-ray data.

Mode	Normalization factor/direction	0.0004 GPa	1.0 GPa	2.23 GPa	3.40 GPa	4.75 GPa	5.20 GPa
Γ1+ Oc	0.0435/(0,0,1)	-0.0046(58)	0.002(21)	-0.038(16)	-0.032(19)	-0.053(77)	-0.0578(26)
Γ1+ O_{ab}	0.0283/(1,1,0)	-0.0324(62)	-0.032(32)	-0.040(24)	-0.022(34)	-0.119(26)	-0.044(44)
Γ1+ C_{DMA}	0.0435/(0,0,1)	0.207(11)	0.197(33)	0.305(24)	0.350(32)	0.324(16)	0.495(38)
Γ4+ O_{ab}	0.0283/(1,-1,0)	-0.0146(66)	-0.006(35)	-0.105(25)	-0.190(36)	-0.123(13)	-0.159(58)
Γ5+ Oc	0.0566/(1,0,0)	-0.1320(61)	-0.134(36)	-0.116(25)	-0.148(37)	-0.164(12)	-0.080(52)
Γ5+ O_{ab}	0.0308/(0,0,1)	-0.2026(64)	-0.267(25)	-0.249(17)	-0.303(21)	-0.2486(86)	-0.338(30)
Γ5+ C_{DMA}	0.0566/(1,0,0)	-1.9553(99)	-2.171(55)	-1.946(43)	-2.034(67)	-1.988(22)	-1.888(75)
A2+ O_{ab}	0.0283/(1,1,0)	-0.5967(62)	-0.477(36)	-0.471(27)	-0.356(33)	-0.302(11)	-0.265(50)
A2+ C_{ab}	0.0400/(1,1,0)	0.4230(95)	0.356(59)	0.331(42)	0.199(56)	0.258(16)	0.125(90)
A3+ O_{ab}	0.0283/(1,-1,0)	-1.9606(65)	-1.982(32)	-1.993(24)	-2.045(34)	-2.050(12)	-2.071(44)
A3+ C_{ab}	0.0400/(1,-1,0)	0.5274(94)	0.430(49)	0.555(36)	0.529(53)	0.483(16)	0.489(71)
A5+ Oc	0.0566/(0,1,0)	-1.8557(62)	-1.886(29)	-2.029(21)	-2.091(26)	-2.089(10)	-2.211(35)
A5+ O_{ab}	0.0308/(0,0,1)	2.1612(63)	2.238(22)	2.228(16)	2.338(18)	2.3157(90)	2.476(25)
A5+ C_{ab}	0.0435(0,0,1)	-0.6488(91)	-0.603(32)	-0.690(25)	-0.718(28)	-0.754(12)	-0.740(40)
A5+ C_c	0.0800/(0,1,0)	-0.4463(91)	-0.511(44)	-0.512(32)	-0.601(35)	-0.575(13)	-0.553(56)
A5+ N_{DMA}	0.0800/(0,1,0)	0.8357(80)	0.855(38)	0.770(26)	0.728(31)	0.703(13)	0.681(48)
A5+ C_{DMA}	0.0566/(0,1,0)	0.531(10)	0.517(57)	0.579(37)	0.591(47)	0.635(18)	0.652(67)
$R_F[I \geq 3\sigma]$ %	-	3.64	6.53	5.31	4.90	4.99	6.01

Table S5. Mode amplitudes refined as function of pressure for the triclinic *P*-1 γ-phase from single crystal x-ray data.

Mode		Normalization factor/direction	7.15 GPa	9.10 GPa
Γ1+	O_c	0.03080/(0,0,1)	0.784(28)	1.081(76)
Γ1+	O_{ab}	0.02000/(1,1,0)	0.611(84)	0.364(29)
Γ1+	C_{DMA}	0.03080/(0,0,1)	-0.312(53)	0.01(11)
Γ2+	O_{ab}	0.02000/(1,1,0)	-1.044(85)	-0.645(30)
Γ3+	O_{ab}	0.02000/(1,-1,0)	1.293(75)	1.325(33)
Γ4+	O_{ab}	0.02000/(1,1,0)	-1.490(73)	-1.281(32)
Γ5+	O_c	0.02830/(1,-1,0)	0.823(98)	0.920(31)
Γ5+	O_c	0.02830/(1,-1,0)	1.826(40)	2.054(33)
Γ5+	O_{ab}	0.03080/(0,0,1)	0.914(32)	0.842(82)
Γ5+	O_{ab}	0.03080/(0,0,1)	-0.084(29)	-0.165(59)
Γ5+	C_{DMA}	0.02830/(1,-1,0)	1.23(17)	1.704(54)
Γ5+	C_{DMA}	0.02830/(1,1,0)	-2.963(76)	-2.662(56)
M2-	N_{DMA}	0.04350/(0,0,1)	-0.836(42)	-1.084(89)
M2-	C_{DMA}	0.03080/(0,0,1)	0.303(51)	0.39(11)
M3-	Cu	0.04350/(0,0,1)	-0.331(05)	-0.46671(80)
M3-	O_c	0.03080/(0,0,1)	-0.462(36)	-0.33043(56)
M3-	O_{ab}	0.02180/(0,0,1)	-0.594(33)	-0.98331(40)
M3-	C_c	0.04350/(0,0,1)	-0.115(51)	-0.46671(80)
M4-	O_{ab}	0.02180/(0,0,1)	-0.070(36)	0.16(10)
M5-	Cu	0.05660/(1,0,0)	-0.062(20)	-0.164(10)
M5-	Cu	0.05660/(0,1,0)	-0.036(11)	-0.0755(80)
M5-	O_c	0.124268/(1,0,0)	0.01(12)	-0.286(33)
M5-	O_c	0.04000/(0,1,0)	-0.017(67)	-0.307(36)
M5-	O_{ab}	0.02000/(1,-1,0)	-0.192(96)	-0.217(37)
M5-	O_{ab}	0.02000/(1,1,0)	0.134(99)	-0.042(44)
M5-	O_{ab}	0.02000/(1,1,0)	0.389(96)	0.571(37)

M5-	O_{ab}	0.02000/(1,-1,0)	0.345(96)	0.034(48)
M5-	C_c	0.05660/(1,0,0)	0.17(16)	-0.163(51)
M5-	C_c	0.05660/(0,1,0)	-0.491(96)	-0.635(53)
M5-	N_{DMA}	0.05660/(1,0,0)	-0.92(14)	-0.962(43)
M5-	N_{DMA}	0.05660/(0,1,0)	0.352(83)	0.291(42)
M5-	C_{DMA}	0.04000/(1,0,0)	1.34(15)	1.254(57)
M5-	C_{DMA}	0.04000/(0,1,0)	0.751(85)	0.942(44)
A1+	O_c	0.03080/(0,0,1)	-0.280(30)	-0.224(74)
A1+	O_{ab}	0.02000/(1,1,0)	0.295(82)	0.249(34)
A1+	C_{ab}	0.02830/(1,1,0)	-0.23(15)	0.548(63)
A1+	C_c	0.04350/(0,0,1)	-0.852(49)	-0.81(13)
A2+	O_{ab}	0.02000/(1,-1,0)	-0.294(77)	-0.195(35)
A2+	C_{ab}	0.02830/(1,-1,0)	-0.32(15)	0.647(62)
A3+	O_{ab}	0.02000/(1,1,0)	3.013(82)	3.179(33)
A3+	C_{ab}	0.02830(1,1,0)	-1.45(13)	-2.056(59)
A4+	O_{ab}	0.02000/(1,1,0)	-0.144(82)	-0.342(34)
A4+	C_{ab}	0.02830/(1,1,0)	-0.84(14)	-1.429(58)
A4+	N_{DMA}	0.04350/(0,0,1)	-0.956(40)	-1.422(99)
A4+	C_{DMA}	0.03080/(0,0,1)	0.828(50)	1.02(12)
A5+	O_c	0.04000/(1,0,0)	-1.442(88)	-1.312(36)
A5+	O_c	0.04000/(0,1,0)	-1.780(51)	-1.825(31)
A5+	O_{ab}	0.02180/(0,0,1)	1.047(32)	1.094(79)
A5+	O_{ab}	0.02180/(0,0,1)	1.593(27)	1.317(71)
A5+	C_{ab}	0.03080/(0,0,1)	-0.787(59)	-1.18(13)
A5+	C_{ab}	0.03080/(0,0,1)	0.128(52)	0.77(11)
A5+	C_c	0.05660/(1,0,0)	-2.57(15)	-2.044(67)
A5+	C_c	0.05660/(0,1,0)	-0.534(94)	-0.890(49)
A5+	N_{DMA}	0.05660/(1,0,0)	0.78(13)	0.585(52)
A5+	N_{DMA}	0.05660/(0,1,0)	0.742(73)	0.788(38)
A5+	C_{DMA}	0.04000/(1,0,0)	-1.09(17)	-0.729(65)
A5+	C_{DMA}	0.04000/(0,1,0)	-0.93(10)	-0.615(51)
Z3-	C_u	0.04350/(0,0,1)	0.001(10)	0.018(31)
Z3-	O_c	0.03080/(0,0,1)	0.038(30)	0.13(12)
Z3-	O_{ab}	0.02180/(0,0,1)	0.008(31)	0.026(85)
Z3-	C_{ab}	0.03080/(0,0,1)	0.229(52)	0.18(14)
Z3-	C_{DMA}	0.03080/(0,0,1)	0.070(50)	0.13(11)
Z4-	O_{ab}	0.02180/(0,0,1)	-0.240(31)	-0.350(92)
Z4-	C_{ab}	0.03080/(0,0,1)	-0.067(54)	-0.05(15)
Z5-	Cu	0.05660/(1,0,0)	0.090(40)	0.021(15)
Z5-	Cu	0.05660/(0,1,0)	0.033(22)	-0.041(10)
Z5-	O_c	0.04000/(1,0,0)	-0.04(10)	0.140(66)
Z5-	O_c	0.04000/(0,1,0)	0.007(64)	0.003(44)
Z5-	O_{ab}	0.02000/(1,1,0)	-0.046(95)	-0.136(44)
Z5-	O_{ab}	0.02000/(1,-1,0)	0.209(97)	0.088(49)
Z5-	O_{ab}	0.02000/(1,-1,0)	0.424(83)	0.379(40)
Z5-	O_{ab}	0.02000/(1,1,0)	0.145(84)	-0.034(45)
Z5-	C_{ab}	0.02830(1,1,0)	0.83(13)	0.599(68)
Z5-	C_{ab}	0.02830(1,-1,0)	-0.36(14)	-0.256(70)
Z5-	C_{ab}	0.02830(1,-1,0)	-1.48(15)	-0.174(76)
Z5-	C_{ab}	0.02830(1,1,0)	-1.32(16)	-0.098(75)
Z5-	C_{DMA}	0.04000/(1,0,0)	0.17(16)	-0.060(60)
Z5-	C_{DMA}	0.04000/(0,1,0)	-0.033(97)	0.023(51)
$R_F[I \geq 3\sigma]$ %		-	8.98	5.56

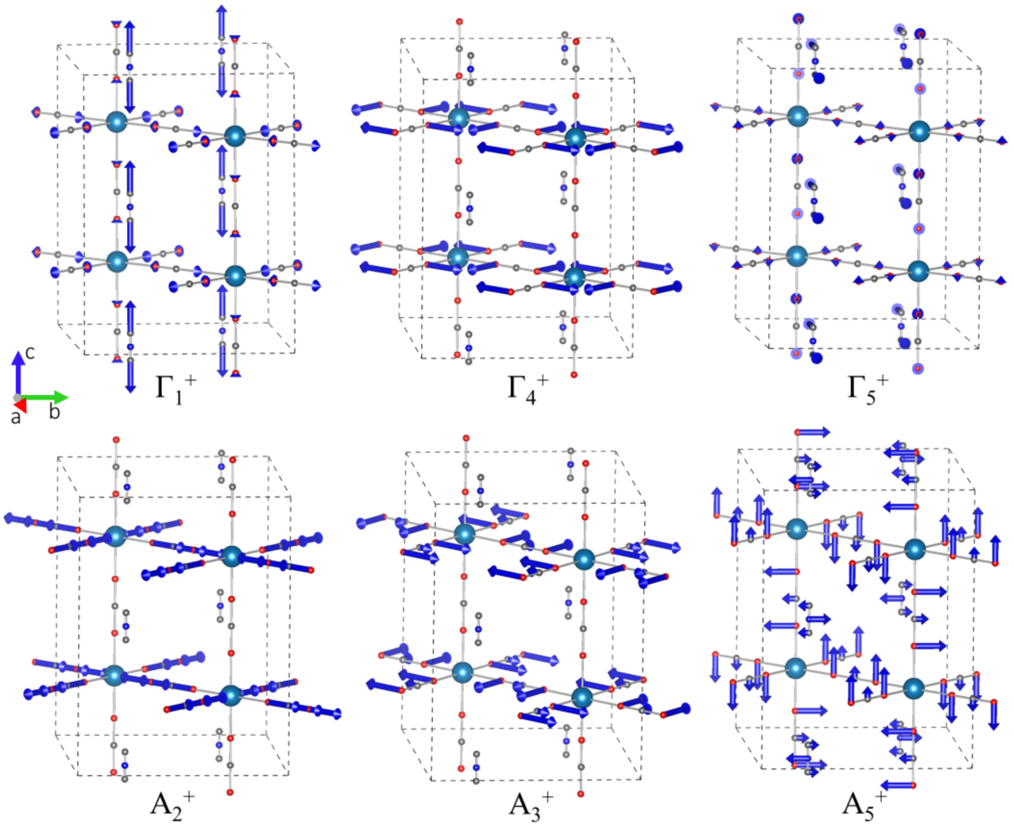

Figure S5. Representation of the active irreducible representation in the monoclinic *I*2/a α-phase of $[(CH_3)_2NH_2]Cu(HCOO)_3$. H atoms were omitted from the analysis.

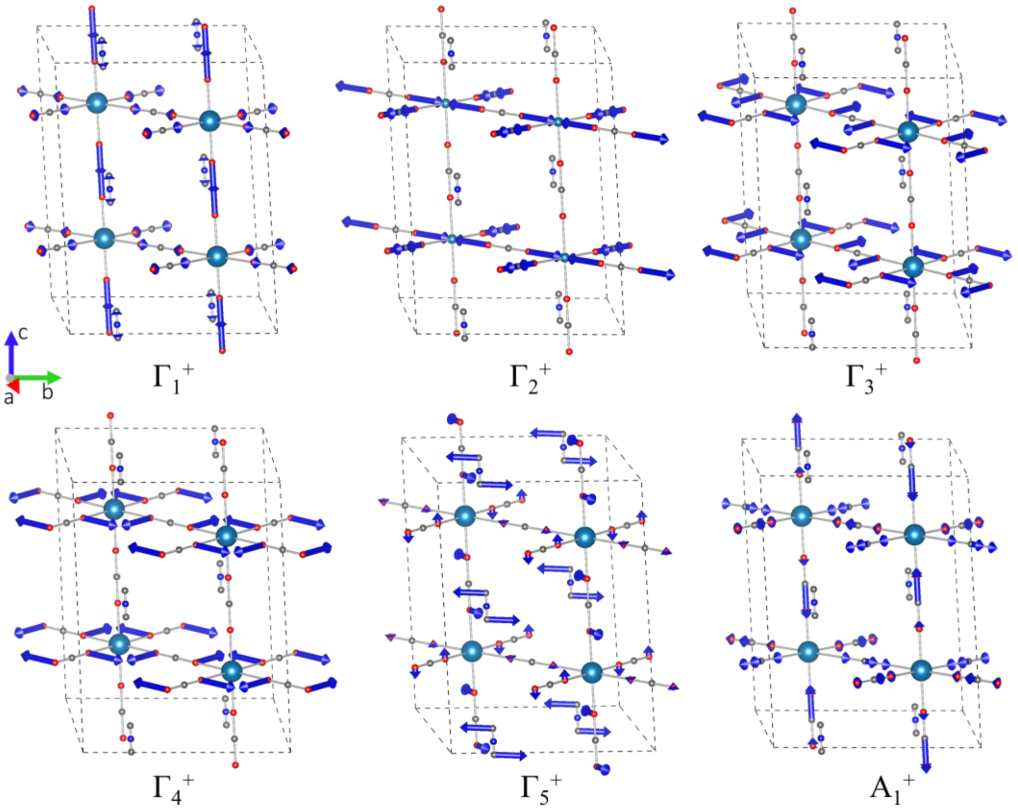

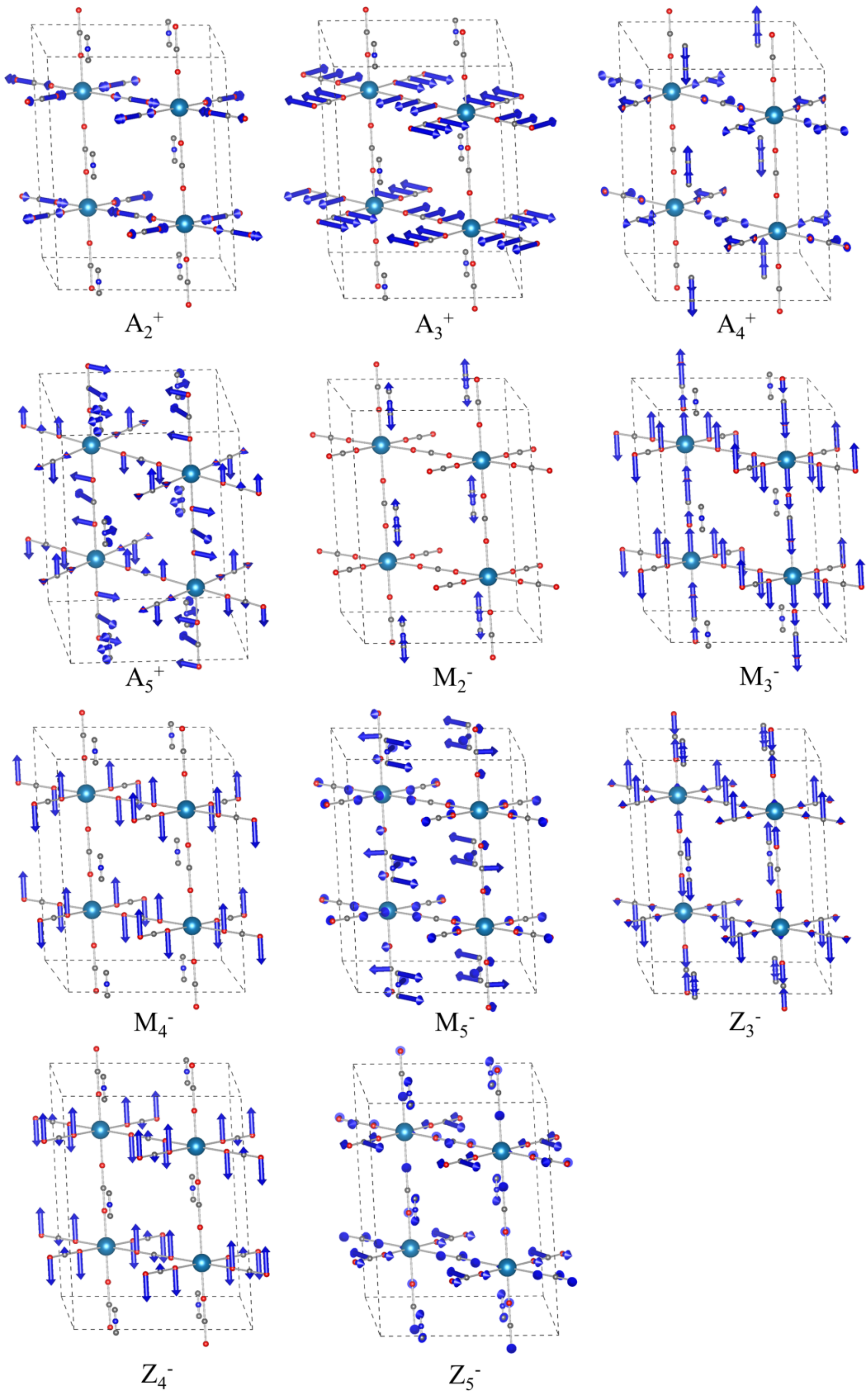

Figure S6. Representation of the active irreducible representation in the triclinic *P*-1 γ-phase of $[(CH_3)_2NH_2]Cu(HCOO)_3$. H atoms were omitted from the analysis.

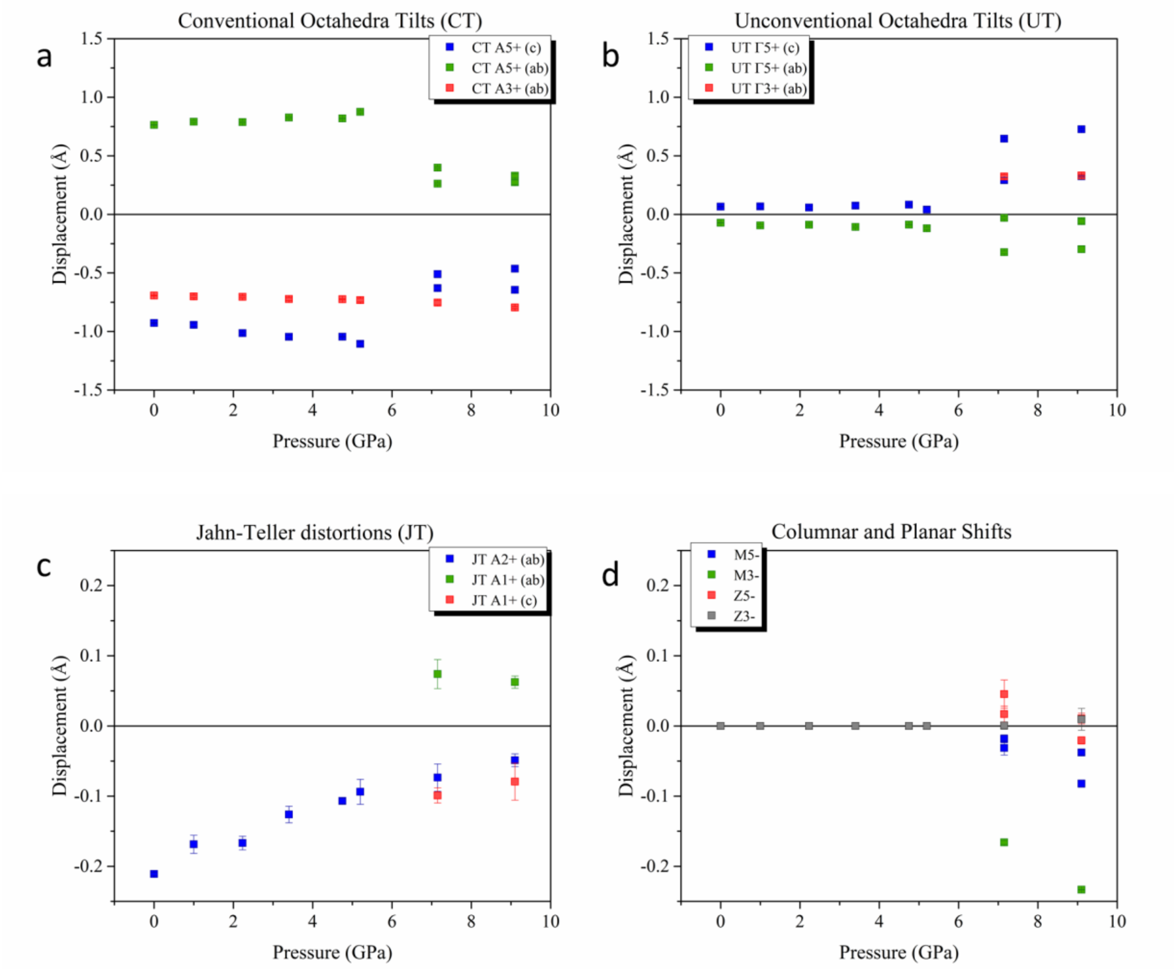

Figure S7. Pressure dependence of the displacement of (a) conventional and (b) unconventional octahedra tilts, (c) Jahn-Teller distortions and (d) Columnar and planar shifts of framework.
